# Latitudinal Cline in Chromosome Numbers of Ice Cod *A. glacialis* (Gadidae) from Northeast Greenland

**DOI:** 10.3390/genes11121515

**Published:** 2020-12-18

**Authors:** Laura Ghigliotti, Jørgen S. Christiansen, Erica Carlig, Davide Di Blasi, Eva Pisano

**Affiliations:** 1 National Research Council of Italy, Institute for the Study of the Anthropic Impacts and the Sustainability of the Marine Environment (IAS) via De Marini 6, 16149 Genoa, Italy; erica.carlig@ias.cnr.it (E.C.); davide.diblasi@ias.cnr.it (D.D.B.); eva.pisano@ias.cnr.it (E.P.); 2 Department of Arctic and Marine Biology, UIT The Arctic University of Norway, NO-9037 Tromsø, Norway; jorgen.s.christiansen@uit.no; 3 Department of Environmental and Marine Biology, Åbo Akademi University, FI-20520 Turku, Finland

**Keywords:** ice cod, karyomorphs, B chromosomes, rDNA, Arctic

## Abstract

The ice cod *Arctogadus glacialis* (Peters, 1872) is one of the few fish species endemic to the Arctic. With a circumpolar distribution, the species is confined to the fjords and shelves of the Arctic seas. Biological information on *A. glacialis* is scarce, with genomic information restricted to microsatellites. Within the frame of the TUNU-Programme: Arctic Ocean Fishes—Diversity, Adaptation and Conservation, we studied *A. glacialis* at the chromosomal level to explore fish diversity and evolutionary aspects. The analysis of over 50 individuals from the Northeast Greenland fjords between latitudes 71°09′ N and 76°42′ N revealed a remarkable intraspecific diversity epitomized by chromosome numbers spanning from 28 to 33, the occurrence of putative B chromosomes, and diversified patterns of distribution of heterochromatin and rDNAs. The number of B chromosomes followed a latitudinal gradient from 0–2 in the north to 2–5 in the south. Considering the benthic and rather stationary life history of this species, the observed chromosomal differences might have arisen independently, possibly driven and/or fostered by the dynamics of repetitive sequences, and are being fixed in relatively isolated fjord populations. The resulting latitudinal cline we observe today might have repercussions on the fate of local populations facing the ongoing climate-driven environmental changes.

## 1. Introduction

The ice cod *Arctogadus glacialis* (Peters, 1872) is one of the few gadid fishes endemic to the Arctic Ocean. The species has historically been reported in Canadian Arctic and Siberian Sea waters, with an apparently disjunct distribution range from the Siberian coast through the Chukchi Sea and the Canadian Arctic to the shelf off Northeast (NE) Greenland. However, verified observations in the European Arctic, supplementing the previously defined distribution, documented a circumpolar continuous distribution of the species [1].

A wide distribution and habitat range have been reported for *A. glacialis*, which seems prevalently associated with cold water masses (−0.6 to 1.5 °C) [2] of fjords and shelves in the coastal Arctic seas. The species has not been recorded in the deeper parts of the Arctic Central Basin [1,3,4].

Key midtrophic species in the high Arctic marine ecosystems, *A. glacialis* channels energy from zooplankton to top predators [5,6]. A broad variety in diet composition indicates an opportunistic pelagic feeding pattern in Northeast Water polynya in NE Greenland [7], while a more benthic behavior of the species is suggested along the Siberian coast [2,8]. In the European Arctic, linkage to the shelf areas is also supported [1], and in NE Greenland fjords, *A. glacialis* is associated with the benthic food web [9].

Compared with other gadid fish inhabiting the Arctic region, *A. glacialis* is still poorly studied [6,10], with genetic information restricted to mtDNA and microsatellites. Based on the mtDNA, a low level of genetic diversity was reported for individuals in NE Greenland [11] and western Canadian waters [12]. Interestingly, *A. glacialis* was found to differ from other northern gadids (namely, *Boreogadus saida*, *Gadus macrocephalus*, *Gadus chalcogrammus*, and *Eleginus gracilis*) in having a highly variable domain containing repeated motifs in the mitochondrial control region [13] and unusual variations of the mtDNA noncoding intergenic T-P spacer, resulting in new stable secondary structures [11]. Microsatellite DNA loci were developed in *A. glacialis* with the aim of aiding population differentiation and species identification [14,15]. Such a DNA-based tool was recently used to remove *A. glacialis* from the dataset in analyses of the circumpolar genetic population structure of the sympatric Arctic gadid *B. saida* [16]. To the best of our knowledge, no information is available on the genetic population structure of *A. glacialis.*

The study of chromosomes, improved by the possibility of physically mapping DNA marker sequences through fluorescence in situ hybridization (FISH), is increasingly being recognized as a powerful tool for describing fish diversity, including population structuring, detection of cryptic species, and evolutionary dynamics (e.g., [17,18,19]). Preliminary data indicated the occurrence of chromosome polymorphism in *A. glacialis*, with chromosome numbers 28, 30, and 32, and variable chromosomal morphology detected in 18 specimens from NE Greenland [20].

Building on those preliminary data, we performed an extensive cytogenetic analysis of the intraspecific chromosomal diversity of *A. glacialis*. Our analysis, of over 50 individuals sampled along the coasts of NE Greenland, confirms the intraspecific chromosome polymorphism in *A. glacialis* and provides a detailed picture of six karyotype variants along a latitudinal cline. The occurrence of supernumerary chromosomes (B chromosomes) is reported for the first time in a gadiform fish and discussed in the context of karyological evolution and ecological frames.

## 2. Materials and Methods

### 2.1. Sampling and Chromosome Preparation

Individuals of *A. glacialis* were obtained from the fjords of NE Greenland during multiple expeditions over a 10-year timeframe (2003–2013), as part of the international TUNU-Programme [21]. Individuals of both sexes were collected from the R/V Helmer Hanssen (formerly R/V Jan Mayen) by bottom trawling along a latitudinal gradient from 71°09′ to 76°43′ N at a depth between 231 and 620 m (sampling details are summarized in Table 1).

The fish were maintained in tanks supplied with fresh, aerated seawater at local ambient temperature and processed on board. Individuals were submitted to mitotic stimulation with concanavalin A, the most efficient among lectins for stimulating cell division in polar fish [22]. Mitotic somatic cells were obtained following protocols for direct chromosome preparations in polar fishes [23]. Briefly, individuals were injected intraperitoneally with colchicine (2 mg colchicine/100 g fish) and later sacrificed with an overdose of anesthetic (MS222). Head kidney and spleen were harvested, and after tissue disaggregation and cell hypotonization, cell suspensions were fixed in 3/1 methanol/acetic acid (*v*/*v*) and stored at −20 °C for later analyses.

Fishes were sampled and treated in accordance with the laws, regulations, and authorization of the government of Greenland (document numbers 28.40.10/2003, 28.40.10/2005, C-10-16, and C-13-16).

### 2.2. Chromosome Analysis and Karyotyping

The chromosome spreads from fixed cells dropped on microscope slides were DAPI (4,4′,6-diamidino-2-phenylindole)-stained and examined with an Olympus BX61 microscope equipped with a SenSys CCD camera (Photometrics). The digital images were processed by CytoVision Genus software (Applied Imaging) or Photoshop (Adobe) or through the open-source scientific image analysis program ImageJ [24] equipped with the Levan plugin [25].

The chromosome number for each individual was determined modally based on a count of 10 to 106 optimal-quality metaphase plates (25 as an average). Individuals whose chromosome preparation provided fewer than 10 high-quality metaphases (from a maximum of 7 microscope slides) were discarded and not included in subsequent analyses. Chromosome morphology was determined on the basis of centromeric position and arm lengths ratio following [26]. The chromosomes were classified as metacentrics (m), submetacentrics (sm), subtelocentrics (st), and acrocentrics (a) and arranged in the karyotype in decreasing order of size.

Further characterization of some chromosomes was obtained by mapping the position of ribosomal genes onto the chromosomes through FISH with a 28S rDNA probe according to [27].

### 2.3. Statistical Analysis

Statistical analyses were performed using R 4.0.2 (R Development Core Team 2020).

The effect of inheritance and mitotic instability on the intraindividual variation of the number of B chromosomes was investigated by calculating the mitotic instability index (*MI*) [28]. In order to avoid bias in the index calculation in individuals with zero as the median number of B chromosomes, the original formula for the *MI* in each individual was modified as follows:

$$MI=\frac{\sum (|M-n_{i}|f_{i})}{\left(M+1\right)\cdot N}$$

where M is the median number of metaphase plates, $n_{i}$ is the number of B chromosomes in the different metaphase plates that do not coincide with M, $f_{i}$ is the number of metaphases of each particular type, and N is the total number of cells analyzed. After having verified the normality and homogeneity of variance through the Shapiro–Wilk and Levene’s tests, a comparative analysis of the *MI* was performed by the one-way ANOVA test and post hoc Tukey’s test (significance at *α* = 0.05).

**Figure 1:**
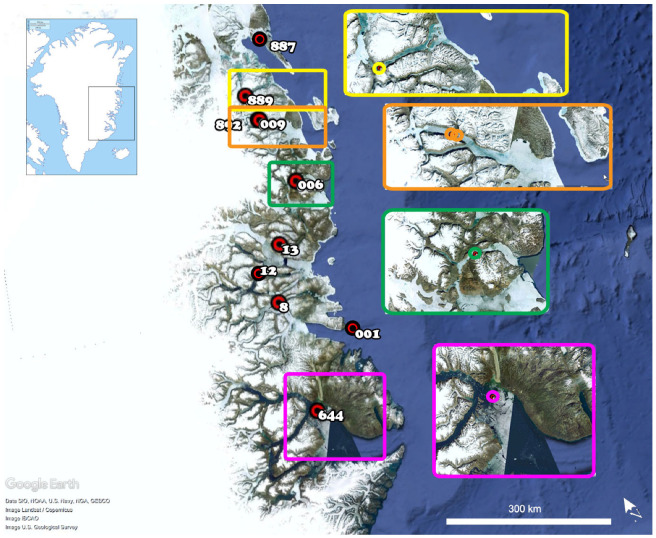
Map of the study area along the coast of Northeast Greenland. Red circles (and numbers) indicate the sampling stations, and colored frames provide a close-up view of the geographical features of the areas considered in the statistical analyses. Yellow frame = area 3; orange frame = area 4; green frame = area 5; pink frame = area 8.

Individual modal numbers of chromosomes were used to test differences in the ploidy level among areas. Well-separated fjord zones with minimal opportunities for exchange between groups were identified (Figure 1) and used as levels for the factor “area”. Stations, and pertaining areas, where only one individual was cytogenetically analyzed (namely, stations nos. 887, 8, and 13) were not considered in subsequent statistical analyses. Station no. 12 was not considered due to the poor quality of the cytogenetic information. The one-way ANOVA test and post hoc Tukey’s test were applied after having verified the normality and homogeneity of variance through the Shapiro-Wilk and Levene’s tests (significance at *α* = 0.05).

## 3. Results

### 3.1. Chromosome Number and Karyotype

Of the 57 individuals studied, 33 specimens produced good-quality chromosome preparations and were included in the subsequent cytogenetic analyses. Irrespective of sex, counts of DAPI-stained metaphases highlighted the occurrence of six different chromosome numbers (range of 28–33).

**Figure 2:**
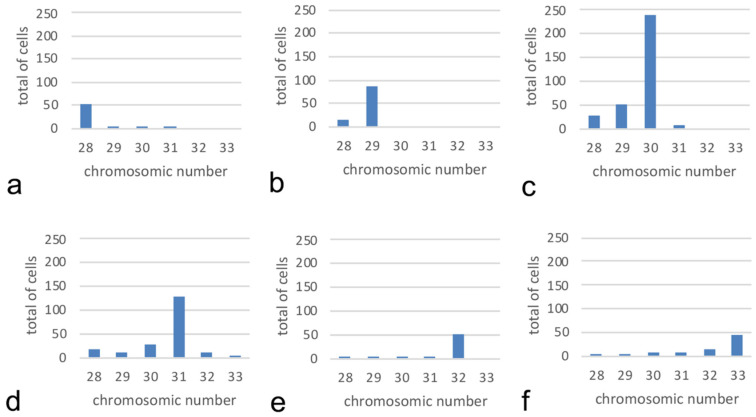
Frequency of chromosome numbers in *A. glacialis* from NE Greenland. Specimens revealed modal chromosome numbers 28 (**a**) (*n* = 3), 29 (**b**) (*n* = 2), 30 (**c**) (*n* = 12), 31 (**d**) (*n* = 10), 32 (**e**) (*n* = 3), and 33 (**f**) (*n* = 3).

Variability was present among cells of the same individual; therefore, the modal diploid number was assigned to individuals. Three specimens (two females and one male) had modal chromosome number 28 for a total of 53 metaphases out of 58 metaphases analyzed (Figure 2a). Two specimens (one female and one male) had modal chromosome number 29 for 86 metaphases out of 100 metaphases analyzed (Figure 2b). Twelve specimens (seven females, four males, and one sexually immature) had modal chromosome number 30 for 231 metaphases out of 367 analyzed (Figure 2c). Ten specimens (four females and six males) had modal chromosome number 31 for 130 metaphases out of 207 analyzed (Figure 2d). Three specimens (one female and two males) had modal chromosome number 32 for 51 metaphases out of 62 analyzed (Figure 2e). Three specimens (one female and two males) had modal chromosome number 33 for 46 metaphases out of 85 analyzed (Figure 2f).

**Figure 3:**
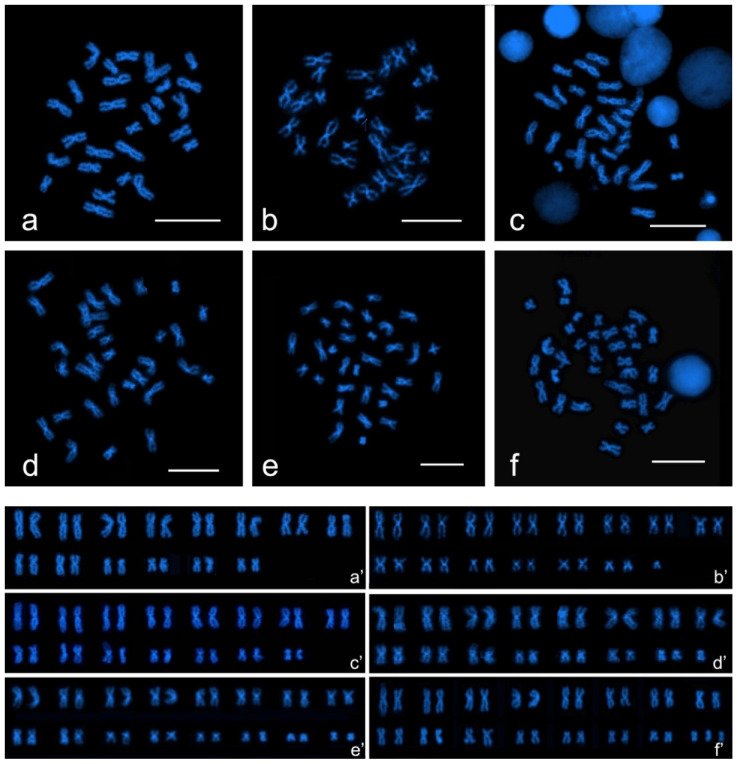
DAPI-stained metaphases and corresponding karyotypic set in *A. glacialis*. Karyomorph A (**a**,**a’**); karyomorph B (**b**,**b’**); karyomorph C (**c**,**c’**); karyomorph D (**d**,**d’**); karyomorph E (**e**,**e’**); karyomorph F (**f**,**f’**). Scale bars = 10 μM.

The six numerical variants corresponded to differences in the macrostructure of chromosomes so that six karyomorphs could be identified. All karyomorphs were entirely composed of two-armed chromosomes, giving a fundamental number (FN) equal to 2 × 2n. Karyomorph A (2n = 28, FN = 56) was made up of 9 pairs of metacentric chromosomes (pairs 1, 3, 4, 5, 6, 7, 8, 10, and 14), 1 pair of submetacentric chromosomes (pair 2), and 4 pairs of chromosomes that were metacentric in some metaphases and submetacentric in others (Figure 3a,a’; Table S1). Karyomorph B (2n = 29, FN = 58) was composed of 9 pairs of metacentric chromosomes (pairs 1, 3, 4, 5, 6, 7, 9, 10, and 14), 2 pairs of submetacentric chromosomes (pairs 2 and 11), 3 pairs of chromosomes that were metacentric in some metaphases and submetacentric in others (pairs 8, 12, and 13), and 1 metacentric chromosome of small size (Figure 3b,b’; Table S1). Karyomorph C (2n = 30, FN = 60) was composed of 14 pairs of metacentric chromosomes and 1 pair of prevalently submetacentric chromosomes (pair 2) (Figure 3c,c’; Table S1). Karyomorph D (2n = 31, FN = 62) was composed of 9 pairs of metacentric chromosomes (pairs 1, 3, 4, 5, 6, 7, 8, 10, and 15), 6 pairs of chromosomes that were metacentric in some metaphases and submetacentric in others (pairs 2, 9, 11, 12, 13, and 14), and 1 metacentric chromosome of small size (Figure 3d,d’; Table S1). Karyomorph E (2n = 32, FN = 64) was composed of 10 pairs of metacentric chromosomes (pairs 1, 3, 4, 5, 6, 7, 8, 9, 12, and 16) and 6 pairs of chromosomes that were metacentric in some metaphases and submetacentric in others (pairs 2, 10, 11, 13, 14, and 15) (Figure 3e,e’; Table S1). Karyomorph F (2n = 33, FN = 66) was composed of 11 pairs of metacentric chromosomes (pairs 1, 2, 3, 4, 5, 6, 8, 10, 14, 15, 16), 5 pairs of prevalently metacentric chromosomes (pairs 7, 9, 11, 12, 13), and 1 metacentric chromosome of small size (Figure 3f,f’; Table S1).

A gradual shift in morphology was observed in the chromosomes of pair 2 that were unambiguously submetacentric when the chromosome number was 28, and metacentric when it was 33; such a change in morphology was accompanied by a decrease of relative length (Table S1). Variability in the position of the centromere, leading to uncertainties in the assignment of chromosomes to morphological classes, was detected across karyomorphs in several pairs of chromosomes of small size. The karyotype variants differ in the occurrence of small metacentric chromosomes ranging from 0 (chromosome number 28) to 5 (chromosome number 33). In the absence of concrete pair markers, and given the relative homogeneity in sizes and shapes between adjacent pairs (especially among pairs 4 to 10), the described karyotypes have to be considered tentative.

### 3.2. Heterochromatin Distribution and Physical Mapping of Major Ribosomal Genes

**Figure 4:**
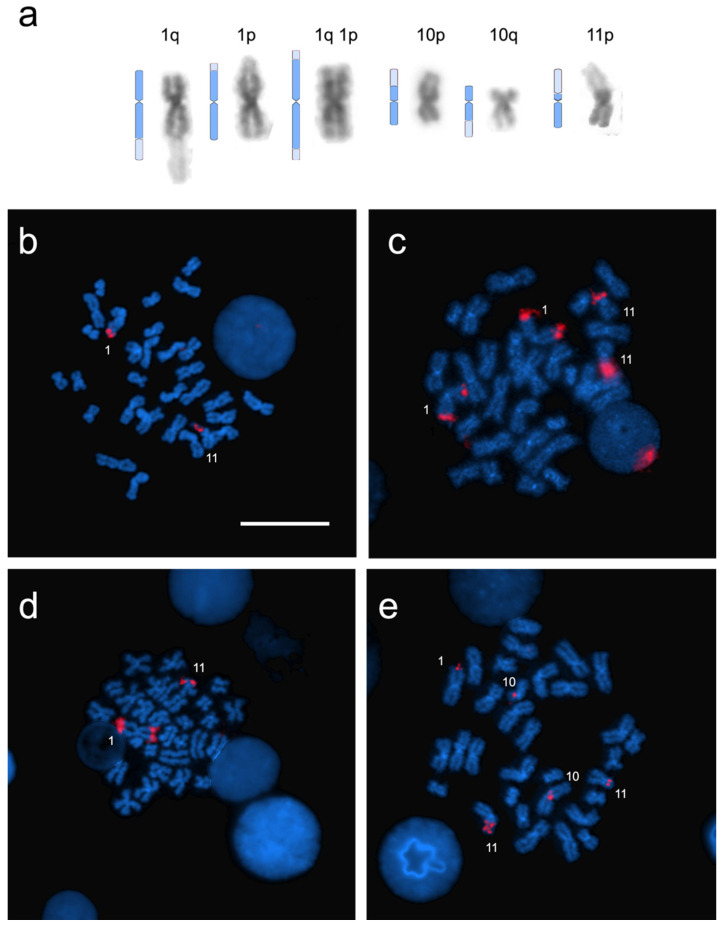
Patterns of dim-DAPI-stained heterochromatic regions and physical mapping of major ribosomal genes on the chromosomes of *A. glacialis.* (**a**) Idiograms of chromosomes with dim-DAPI-stained regions; (**b**) strong 28S rDNA hybridization signals on one of the homologues of pair 1 (at the telomeric position on the q arms) and pair 11 (along the entire p arms); (**c**) signals on pair 1 (at the telomeric position on the p and q arms of both homologues) and size heteromorphism of signals on the p arms of pair 11; (**d**) signals on one of the homologues of pair 1 (at the telomeric position on the p and q arms); (**e**) rDNA signals on pair 1 (at the telomeric position on the q arms of one of the homologues), pair 10 (at the pericentromeric position on the p arms of a heteromorphic pair of chromosomes), and pair 11 (along the entire p arms of heteromorphic chromosomes). Scale bar = 10 μM.

Dim-DAPI-stained heterochromatic regions were observed at the telomeric position on the p and/or q arms of pair 1, p and/or q arms of pair 10, and p arms of pair 11. Those dim-DAPI-stained regions were sometimes very large in their extent, changing the chromosome typing from submetacentric to metacentric and occasionally making the p arm longer than the q arm. The extent of the dim-DAPI-stained regions usually differed between the homologues, leading to chromosome heteromorphism within the pair. The distribution of the dim-DAPI-stained regions differed among individuals, and multiple randomly combined patterns were observed (Figure 4a).

The physical mapping of 28S rDNA resulted in multiple chromosomal locations. Major ribosomal gene signals were often detected corresponding to short dim-DAPI-stained telomeric regions on the q arm of the chromosomes of pair 1 (Figure 4a,e). Clusters of major rDNA were also often found on a large portion of the p arms of the chromosomes of pair 11; the extent of the ribosomal gene-bearing region was usually different between the homologues, leading to heteromorphism of the homologues in pair 11 (Figure 4a–e). Occasionally, the 28S rDNA probe mapped at the telomeric position of both the p and q arms of pair 1 (Figure 4b,c) and the p arm of pair 10 at the interstitial position (Figure 4e). In this latter case, the observed major rDNA signals were usually small and not corresponding to the entire dim-DAPI-stained heterochromatic region. The major ribosomal gene sites differed among specimens, resulting in a number of signals ranging from one to eight per individual. No correspondence was detected between 28S rDNA patterns and chromosome numbers (i.e., the various patterns occurred equally across karyomorphs).

### 3.3. Mitotic Instability, Chromosome Number, and Geographical Distribution

Statistical analyses were conducted on a subsample of 30 individuals from four areas: area 3 (*n* = 10), area 4 (*n* = 5), area 5 (*n* = 5), and area 8 (*n* = 10).

The variability in the diploid number among cells of the same individual, ascribable to the presence of small metacentric chromosomes in a range of 0–5, was investigated in a sample to test the mitotic instability of those chromosomes. Of the 30 individuals analyzed, 28 showed mitotic instability and an MI ranging between 0.02 and 0.30. The MI did not differ significantly between groups of individuals from different areas (Df = 3, *F* value = 0.671, *p* = 0.577).

The analysis of the spatial distribution of karyomorphs highlighted differences in the frequency of occurrence of the karyotype variants within the study area (summary of the available data per specimen in Table S2). Lower modal chromosome numbers (28 and 29), corresponding to small B chromosomes in the range of 0–2, occur in the northernmost part of the study area (area 3), where 30 is the most frequent modal number of chromosomes. Higher modal chromosome numbers and number of B chromosomes ranging from 0 to 5 are common in the southernmost area (area 8), with 31 as the most frequent modal number. A mixture of karyomorphs, with no prevailing one, was found in areas 4 and 5. The chromosome numbers were tested against the areas. The Shapiro–Wilk and Levene’s tests resulted in three out of the four groups presenting normal distribution and homogeneous variances among groups. The ANOVA test revealed significant differences among the areas (Df = 3, *F* value = 4.285, *p* = 0.0139), post hoc Tukey’s test identified a significant difference between area 3 (2n = 29.5 ± 0.85 SD) and area 8 (2n = 31.3 ± 1.06 SD), and area 4 (2n = 31.0 ± 1.22 SD) and area 5 (2n = 30.4 ± 1.82 SD) were not significantly different from any of the others.

## 4. Discussion

Among the Arctic gadids, *A. glacialis* is the least studied species [10], and this study is the first to provide an in-depth characterization of the species’ cytogenetic features. The analysis, conducted on over 50 individuals collected from fjord systems of NE Greenland, revealed a remarkable intraspecific diversity, epitomized by diploid number (2n) spanning from 28 to 33. Different ploidy levels were coupled with differences in the chromosome macrostructure, leading to the identification of six different karyomorphs.

While karyotype stasis, that is the absence of changes in the chromosome number and genomic structure [29], is a recurrent condition in several taxa, including fishes (e.g., [30,31,32]), the intraspecific karyotype variability has been described in a number of species, from both freshwater (e.g., [18,33,34,35]) and marine (e.g., [36,37,38]) habitats. In most cases, the observed variability in the 2n and chromosome macrostructure is explained by advocating Robertsonian rearrangements. These mechanisms of chromosomal reshaping, fissions, and/or fusions, alter the 2n, while the fundamental number (FN) remains the same. Within Gadidae, intraspecific polymorphism connected with Robertsonian rearrangements was observed in Atlantic cod *Gadus morhua* [39]. However, the differences observed in *A. glacialis*, involving both the number of chromosomes and FN, cannot be explained by rearrangements of the Robertsonian type. The overall size and morphology of chromosomes of pairs 1 to 14 are quite conserved among karyomorphs. The only remarkable morphological change is the shift of chromosomes of pair 2 from submetacentric to metacentric when moving from the lowest chromosome number (28) to the highest (33). Such a morphological change is coupled with a slight decrease in the relative size of the chromosome. Breaks of the chromosomes of pair 2 might have progressively changed their morphology and size, while originating small-sized chromosomes as by-product. This leads us to consider the hypothesis that the diversity in the chromosome number observed among *A. glacialis* would be due to the occurrence of B chromosomes. B chromosomes are additional elements to the regular chromosome set found in some but not all individuals within a population, and undergo non-Mendelian inheritance. In *A. glacialis*, two-armed chromosomes of small size (the smallest of the karyotype) occur in the range of 0–5 between individuals. The distribution of these small elements shows variation in the number per cell (mosaic distribution), possibly reflecting the absence of the Mendelian segregation pattern for these chromosomes. A non-Mendelian mechanism of inheritance of the putative B chromosomes observed in *A. glacialis* is supported by the proportion of individuals analyzed showing mitotic instability (93%) and by the values of individual mitotic instability indexes ranging from 0.22 to 0.30. The occurrence of B chromosomes has been reported in 114 fish species (http://www.bchrom.csic.es/; [40]). The presence of B chromosomes is remarkably recurrent in the fish orders Characiformes and Siluriformes, both characterized by the tendency to retain two-armed chromosomes, which is also the most frequent morphology in their supernumerary chromosomes [41,42,43]. Evidence of the occurrence of meiotic drive involving B chromosomes in fish karyotypes with high two-armed elements has been presented, supporting the association between karyotypes with a high number of two-armed chromosomes and B chromosomes [44]. Although B chromosomes have never been reported in Gadiformes [45], the karyotypic features of *A. glacialis*, and specifically the occurrence of only metacentric/submetacentric chromosomes, are coherent with the hypothesized association between the prevalently two-armed karyotypes and B chromosomes. B chromosomes are assumed to have been derived from standard genomic elements from the same or a different species, and are prone to accumulating repetitive DNA sequences during their evolution [46]. Traditionally, they are described as rich in heterochromatin, gene poor, and composed of repeated DNA sequences. Occasionally, ribosomal genes are found on B chromosomes in fish species [47,48]. The uneven DAPI staining of *A. glacialis* small B chromosomes, characterized by DAPI-positive telomeric regions and dim-DAPI-stained arms and centromere, might indicate extensive heterochromatic composition of these chromosomes that, however, do not carry major ribosomal DNA sequences. Further studies would be necessary to investigate the nature of the chromatin for *A. glacialis* B chromosomes, including molecular cytogenetic approaches and omics techniques, and to ascertain the origin of these genomic elements.

Regardless of their mechanism of origin, the occurrence of B chromosomes in *A. glacialis* might have interesting functional repercussions, whose consideration in future studies might help in interpreting their presence in an ecological frame. Although classically considered selfish elements, their influence on the cell biology has recently been reconsidered. Owing to omics analyses, B chromosomes have been demonstrated not to be genetically silent, but possibly containing transcriptionally active sequences (reviewed in [49,50]). In some species, the appearance of B chromosomes was apparently favored by adverse environmental conditions, such as pollution, and an adaptive value of these chromosomes was suggested [51]. In general, given that transcription factors, miRNAs, and other ncRNA regulators have been found in B chromosomes, it can be hypothesized that the expression of those B-sequences might trigger modifications of the A-genome expression, ultimately leading to changes with potential repercussion on the fitness of individuals.

Another aspect of *A. glacialis* karyology that deserves attention is the remarkable level of interindividual chromosomal variability detected in the distribution of dim-DAPI-stained heterochromatin, partially corresponding to major rDNA-bearing chromatin. In general, this is not surprising since these sequences, organized in the genome in multiple clusters of widely variable sizes, segregate randomly and independently, making the number and location of detectable major rDNA loci variable also at an intraspecific level [52]. Moreover, diversified patterns of distribution of heterochromatin blocks and rDNAs on different chromosomes might be related to the dynamics of dispersion of repetitive sequences, often associated with transposable elements (TE) [53]. Transposons have been reported to occur in spacer regions of rDNA sequences, foster the spread of rDNAs in functional copies or pseudogenes, affect the rate of recombination, and ultimately lead to the divergence of karyotypes [54,55,56,57]. In this sense, the diversified patterns of repetitive DNA sequences found in the genome of *A. glacialis* might entail a high degree of genomic plasticity for the species, which might translate into variations of phenotype and fitness.

Remarkably, in *A. glacialis* when analyzing the karyologic information on a geographical scale, the frequency of occurrence of putative B chromosomes differed between sampling areas with the number of B chromosomes following a latitudinal cline, 0–2 B chromosomes in the northernmost area to 2–5 B chromosomes in the southernmost area. The differential frequency of B chromosomes between areas was statistically significant. Considering the benthic and rather stationary life history of this species [9], and the fragmented NE Greenland coastline characterized by a large number of secluded fjords and different physiochemical water properties, we hypothesize that a degree of genomic differentiation might have arisen independently, possibly driven and/or fostered by the dynamics of repetitive sequences, and might have been fixed in relatively isolated fjord populations due to limited connectivity. Further information on the life history and population structuring of *A. glacialis* in NE Greenland is needed to support this hypothesis.

Overall, the genome plasticity observed in *A. glacialis*, testified by the occurrence of B chromosomes and diversified patterns of ribosomal DNA, seems to be a favorable character of an Arctic species, potentially providing genomic tools to face the challenges posed by the ongoing pronounced climate-driven environmental changes.

## Figures and Tables

**Table 1:** Bottom trawl locations during the TUNU expeditions to Northeast Greenland. Numbers of analyzed male and female *Arctogadus glacialis* are shown for given locations. The background color makes it easier to read the table.

Expedition	Year	Station ID	Latitude N	Longitude W	Depth	Males	Females
TUNU I	2003	887	76°43′	19°17′	231	1	0
TUNU I	2003	889	75°58′	21°41′	234	5	6
TUNU I	2003	892	75°33′	21°39′	571	2	3
TUNU II	2005	644	71°09′	24°57′	483	11	8
TUNU IV	2010	8	72°53′	24°40′	327	0	1
TUNU IV	2010	12	73°25′	25°17′	440	2	1
TUNU IV	2010	13	73°42′	23°28′	232	0	1
TUNU V	2013	001	72°00′	21°01′	466	0	1
TUNU V	2013	006	74°27′	21°10′	316	5	4
TUNU V	2013	009	75°33′	21°38′	620	2	1
**Specimen total**						**28**	**26**

## Supplementary Materials

The following is available online at https://www.mdpi.com/2073-4425/11/12/1515/s1: Table S1: Synthesis of cytogenetic information on karyomorphs of *Arctogadus glacialis*; Table S2: Summary of the available data per specimen.
