# Supplementary material for: Latitudinal Cline in Chromosome Numbers of Ice Cod A. glacialis (Gadidae) from Northeast Greenland
Source: Genes (Basel). 2020 Dec 18;11(12):1515. doi: 10.3390/genes11121515 (PMC7766978; doi:10.3390/genes11121515)
Supplement: Supplementary file 1 [file genes-11-01515-s001.pdf]

Table S1 Synthesis of cytogenetic information on karyomorphs of *Arctogadus glacialis*. CI = centromeric index (mean ± standard deviation); AR = arm ratio (mean ± standard deviation); CT = chromosome type (determined following Levan et al. 1964); RL = relative length (mean ± standard deviation).

	2n=28				2n=29				2n=30			
Pair nr.	CI	AR	CT	RL	CI	AR	CT	RL	CI	AR	CT	RL
1	47.22 (± 1.58)	1.12 (± 0.07)	m	5.01 (± 0.09)	48,23 (± 1.63)	1.08 (± 0.07)	m	5.15 (± 0.47)	49,28 (± 1.02)	1.03 (± 0.04)	m	4.67 (± 0.31)
	46,45 (± 2.79)	1.16 (± 0.13)	m	4.65 (± 0.09)	46,48 (± 5.16)	1.17 (± 0.26)	m	4.88 (± 0.54)	47,29 (± 1.11)	1.03 (± 0.13)	m	4.52 (± 0.15)
2	36.35 (± 0.41)	1.75 (± 0.03)	sm	4.53 (± 0.18)	31,81 (± 3.53)	2.17 (± 0.38)	sm	4.72 (± 0.43)	42,67 (± 14.10)	1.78 (± 0.38)	sm (m)	4.19 (± 0.27)
	34.36 (± 1.14)	1.91 (± 0.10)	sm	4.46 (± 0.20)	35,14 (± 0.32)	1.85 (± 0.03)	sm	4.53 (± 0.34)	42,84 (± 13.33)	1.74 (± 0.19)	sm (m)	4.02 (± 0.35)
3	45.01 (± 3.89)	1.23 (± 0.19)	m	4.33 (± 0.34)	47,53 (±2.69)	1.11 (± 0.12)	m	4.55 (± 0.52)	49,70 (± 3.75)	1.11 (± 0.11)	m	4.49 (± 0.09)
	46.83 (± 1.76)	1.14 (± 0.08)	m	4.38 (± 0.08)	47,86(± 2.10)	1.09 (± 0.09)	m	4.58 (± 0.44)	48,20 (± 3.94)	1.11 (± 0.16)	m	4.40 (± 0.12)
4	47.38 (± 2.00)	1.11 (± 0.09)	m	4.11 (± 0.25)	44,57 (± 2.15)	1.25 (± 0.11)	m	4.51 (± 0.30)	50,06 (± 4.33)	1.12 (± 0.13)	m	4.13 (± 0.24)
	46.39 (± 2.46)	1.16 (± 0.12)	m	4.037 (± 0.07)	46,96 (± 1.23)	1.13 (± 0.06)	m	4.25 (± 0.08)	48,59 (± 4.20)	1.12 (± 0.15)	m	4.02 (± 0.21)
5	45.28 (± 1.90)	1.21 (± 0.09)	m	3.99 (± 0.16)	49,45 (±0.69)	1.02 (± 0.03)	m	4.10 (± 0.15)	49,53 (± 4.94)	1.16 (± 0.13)	m	3.90 (± 0.13)
	45.88 (± 1.88)	1.18 (± 0.09)	m	3.93 (± 0.18)	46,68 (± 2.55)	1.15 (± 0.11)	m	4.01 (± 0.26)	46,42 (± 7.03)	1.30 (± 0.20)	m	3.83 (± 0.04)
6	47.56 (± 2.45)	1.11 (± 0.11)	m	4.08 (± 0.04)	47,03 (± 2.56)	1.13 (± 0.11)	m	3.71 (± 0.20)	49,71 (± 3.91)	1.12 (± 0.09)	m	3.71 (± 0.26)
	45.46 (± 5.12)	1.22 (± 0.26)	m	3.84 (± 0.20)	46,47 (±0.97)	1.15 (± 0.05)	m	3.44 (± 0.36)	48,05 (± 6.25)	1.22 (± 0.20)	m	3.76 (± 0.21)
7	47.96 (± 1.13)	1.09 (± 0.05)	m	3.90 (± 0.12)	41,92 (±0.82)	1.39 (± 0.05)	m	3.77 (± 0.44)	49,43 (± 4.91)	1.17 (± 0.11)	m	3.74 (± 0.22)
	46.86 (± 1.86)	1.14 (± 0.08)	m	3.56 (± 0.08)	42,44 (± 2.33)	1.36 (± 0.13)	m	3.91 (± 0.08)	49,91 (± 5.48)	1.18 (± 0.15)	m	3.82 (± 0.07)
8	44.19 (± 1.66)	1.27 (± 0.09)	m	3.70 (± 0.05)	36,59 (± 2.06)	1.74 (± 0.16)	sm (m)	3.74 (± 0.22)	44,66 (± 4.49)	1.26 (± 0.21)	m	3.43 (± 0.23)
	42.77 (± 4.13)	1.35 (± 0.22)	m	3.61 (± 0.13)	36,90 (± 2.36)	1.72 (± 0.18)	sm (m)	3.70 (± 0.10)	46,10 (± 4.19)	1.21 (± 0.16)	m	3.44 (± 0.33)
9	43.16 (± 3.32)	1.33 (± 0.19)	m	3.71 (± 0.03)	46,82 (± 2.50)	1.14 (± 0.12)	m	3.41 (± 0.35)	47,55 (± 7.67)	1.29 (± 0.21)	m	3.36 (± 0.10)
	39.79 (± 3.39)	1.53 (± 0.21)	m (sm)	3.61 (± 0.08)	46,47 (± 3.73)	1.16 (± 0.18)	m	3.36 (± 0.46)	48,07 (± 7.21)	1.27 (± 0.18)	m	3.29 (± 0.08)
10	47.10 (± 2.37)	1.13 (± 0.10)	m	3.52 (± 0.21)	45,59 (± 1.04)	1.19 (± 0.05)	m	3.41 (± 0.51)	50,02 (± 6.22)	1.21 (± 0.18)	m	3.21 (± 0.24)
	47.56 (± 1.33)	1.10 (± 0.06)	m	3.34 (± 0.29)	43,47 (± 2.79)	1.31 (± 0.15)	m	3.13 (± 0.27)	48,60 (± 7.16)	1.27 (± 0.16)	m	3.18 (± 0.22)
11	35.84 (± 2.78)	1.80 (± 0.21)	sm (m)	2.68 (± 0.20)	30,35 (± 1.59)	2.30 (± 0.17)	sm	2.39 (± 0.24)	58,50 (± 3.42)	0.99 (± 0.47)	m	3.14 (± 0.47)
	35.18 (± 1.81)	1.85 (± 0.14)	sm (m)	2.66 (± 0.15)	32,03 (± 1.24)	2.12 (± 0.12)	sm	2.40 (± 0.19)	45,58 (± 10.09)	1.44 (± 0.13)	m	2.44 (± 0.30)
12	33.13 (± 6.21)	2.08 (± 0.52)	sm (m)	2.42 (± 0.17)	38,21 (± 2.67)	1.63 (± 0.19)	m (sm)	2.05 (± 0.07)	46,20 (± 10.07)	1.47 (± 0.10)	m	2.41 (± 0.22)
	35.80 (± 5.27)	1.83 (± 0.41)	sm (m)	2.38 (± 0.06)	37,48 (± 3.58)	1.68 (± 0.26)	m (sm)	2.16 (± 0.09)	45,38 (± 9.12)	1.44 (± 0.13)	m	2.46 (± 0.19)
13	42.85 (± 5.41)	1.36 (± 0.29)	m (sm)	2.41 (± 0.09)	40,27 (±5.25)	1.51 (± 0.35)	m (sm)	2.18 (± 0.04)	44,77 (± 6.82)	1.32 (± 0.33)	m	2.26 (± 0.29)
	42.13 (± 5.86)	1.40 (± 0.31)	m (sm)	2.33 (± 0.07)	40,54 (± 5.03)	1.49 (± 0.30)	m (sm)	2.15 (± 0.20)	43,69 (± 7.52)	1.42 (± 0.22)	m	2.21 (± 0.11)
14	43.33 (± 2.33)	1.31 (± 0.13)	m	2.48 (± 0.20)	39,97 (± 2.15)	1.35 (± 0.07)	m	2.07 (± 0.21)	47,81 (± 6.36)	1.25 (± 0.14)	m	2.21 (± 0.19)
	42.25 (± 3.71)	1.38 (± 0.20)	m	2.31 (± 0.15)	42,65 (± 1.27)	1.51 (± 0.14)	m	2.14 (± 0.15)	47,16 (± 5.79)	1.24 (± 0.15)	m	2.11 (± 0.11)
15					44,90 (± 2.39)	1.23 (± 0.12)	m	1.56 (± 0.18)	45,82 (± 1.57)	1.10 (± 0.18)	m	1.88 (± 0.22)
									48,11 (± 1.03)	1.03 (± 0.09)	m	1.74 (± 0.10)

Table 2 (continued)

	2n=31				2n=32				2n=33			
Pair nr.	CI	AR	CT	RL	CI	AR	CT	RL	CI	AR	CT	RL
1	0.45 (± 0.07)	1.25 (± 0.40)	m	4.49 (± 0.19)	0.44 (± 0.03)	1.30 (± 0.14)	m	4.33 (± 0.44)	0.47 (± 0.04)	1.13 (± 0.19)	m	4.98 (± 0.86)
	0.46 (± 0.02)	1.15 (± 0.08)	m	4.33 (± 0.13)	0.45 (± 0.02)	1.22 (± 0.08)	m	4.21 (± 0.43)	0.47 (± 0.03)	1.14 (± 0.13)	m	4.35 (± 0.31)
2	0.41 (± 0.04)	1.47 (± 0.23)	m (sm)	4.29 (± 0.24)	0.39 (± 0.02)	1.59 (± 0.16)	m (sm)	4.00 (± 0.31)	0.39 (± 0.01)	1.58 (± 0.09)	m	4.04 (± 0.24)
	0.38 (± 0.01)	1.62 (± 0.10)	m (sm)	4.32 (± 0.18)	0.39 (± 0.02)	1.57 (± 0.14)	m (sm)	4.03 (± 0.26)	0.39 (± 0.02)	1.56 (± 0.12)	m	3.99 (± 0.27)
3	0.44 (± 0.01)	1.26 (± 0.05)	m	4.20 (± 0.27)	0.46 (± 0.03)	1.17 (± 0.13)	m	4.20 (± 0.31)	0.45 (± 0.04)	1.22 (± 0.18)	m	4.06 (± 0.45)
	0.45 (± 0.01)	1.23 (± 0.06)	m	4.29 (± 0.23)	0.47 (± 0.02)	1.14 (± 0.11)	m	4.28 (± 0.22)	0.46 (± 0.05)	1.21 (± 0.26)	m	4.11 (± 0.41)
4	0.46 (± 0.02)	1.19 (± 0.10)	m	4.00 (± 0.14)	0.49 (± 0.00)	1.06 (± 0.02)	m	3.87 (± 0.10)	0.47 (± 0.01)	1.13 (± 0.03)	m	3.86 (± 0.02)
	0.47 (± 0.02)	1.14 (± 0.09)	m	4.08 (± 0.03)	0.49 (± 0.00)	1.04 (± 0.01)	m	3.85 (± 0.14)	0.48 (± 0.00)	1.10 (± 0.01)	m	3.87 (± 0.08)
5	0.46 (± 0.02)	1.16 (± 0.08)	m	3.87 (± 0.14)	0.48 (± 0.01)	1.08 (± 0.06)	m	3.80 (± 0.12)	0.45 (± 0.03)	1.24 (± 0.17)	m	3.65 (± 0.14)
	0.47 (± 0.02)	1.14 (± 0.11)	m	3.82 (± 0.15)	0.49 (± 0.01)	1.06 (± 0.03)	m	3.80 (± 0.13)	0.46 (± 0.03)	1.18 (± 0.13)	m	3.65 (± 0.13)
6	0.46 (± 0.02)	1.21 (± 0.11)	m	3.71 (± 0.18)	0.46 (± 0.01)	1.17 (± 0.05)	m	3.56 (± 0.09)	0.47 (± 0.03)	1.14 (± 0.13)	m	3.66 (± 0.09)
	0.46 (± 0.03)	1.20 (± 0.15)	m	3.70 (± 0.36)	0.45 (± 0.01)	1.20 (± 0.07)	m	3.59 (± 0.10)	0.47 (± 0.04)	1.13 (± 0.16)	m	3.69 (± 0.07)
7	0.44 (± 0.04)	1.33 (± 0.33)	m	3.39 (± 0.14)	0.44 (± 0.03)	1.26 (± 0.15)	m	3.44 (± 0.17)	0.43 (± 0.03)	1.32 (± 0.18)	m (sm)	3.43 (± 0.28)
	0.44 (± 0.02)	1.28 (± 0.13)	m	3.43 (± 0.16)	0.44 (± 0.03)	1.29 (± 0.19)	m	3.44 (± 0.16)	0.43 (± 0.03)	1.32 (± 0.17)	m	3.40 (± 0.24)
8	0.42 (± 0.04)	1.49 (± 0.12)	m	3.29 (± 0.15)	0.42 (± 0.03)	1.41 (± 0.18)	m	3.41 (± 0.16)	0.42 (± 0.05)	1.38 (± 0.28)	m	3.42 (± 0.14)
	0.43 (± 0.03)	1.40 (± 0.17)	m	3.18 (± 0.16)	0.43 (± 0.02)	1.31 (± 0.10)	m	3.37 (± 0.08)	0.43 (± 0.05)	1.32 (± 0.26)	m	3.41 (± 0.20)
9	0.42 (± 0.08)	1.51 (± 0.28)	m (sm)	3.23 (± 0.18)	0.45 (± 0.03)	1.24 (± 0.16)	m	3.18 (± 0.21)	0.45 (± 0.02)	1.22 (± 0.09)	m	3.19 (± 0.20)
	0.41 (± 0.07)	1.57 (± 0.36)	m (sm)	3.20 (± 0.18)	0.45 (± 0.03)	1.23 (± 0.16)	m	3.20 (± 0.23)	0.45 (± 0.02)	1.22 (± 0.11)	m (sm)	3.17 (± 0.20)
10	0.46 (± 0.02)	1.23 (± 0.02)	m	3.19 (± 0.19)	0.49 (± 0.06)	1.06 (± 0.26)	m	3.43 (± 0.55)	0.44 (± 0.05)	1.28 (± 0.27)	m	2.81 (± 0.19)
	0.46 (± 0.03)	1.21 (± 0.12)	m	3.05 (± 0.13)	0.41 (± 0.08)	1.49 (± 0.50)	m (sm)	2.97 (± 0.20)	0.44 (± 0.05)	1.28 (± 0.30)	m	2.79 (± 0.14)
11	0.47 (± 0.11)	1.20 (± 0.57)	m (sm)	3.17 (± 0.55)	0.42 (± 0.07)	1.43 (± 0.42)	m (sm)	2.95 (± 0.47)	0.39 (± 0.04)	1.58 (± 0.27)	m (sm)	2.69 (± 0.35)
	0.45 (± 0.10)	1.30 (± 0.60)	m (sm)	2.88 (± 0.28)	0.35 (± 0.03)	1.85 (± 0.24)	sm (m)	2.71 (± 0.10)	0.39 (± 0.04)	1.56 (± 0.24)	m (sm)	2.70 (± 0.36)
12	0.37 (± 0.05)	1.72 (± 0.37)	sm (m)	2.368 (± 0.21)	0.42 (± 0.04)	1.39 (± 0.26)	m	2.65 (± 0.28)	0.41 (± 0.03)	1.45 (± 0.19)	m	2.24 (± 0.11)
	0.37 (± 0.04)	1.70 (± 0.31)	sm (m)	2.44 (± 0.16)	0.42 (± 0.05)	1.39 (± 0.30)	m	2.60 (± 0.10)	0.41 (± 0.05)	1.46 (± 0.27)	sm (m)	2.24 (± 0.15)
13	0.41 (± 0.06)	1.46 (± 0.37)	m (sm)	2.147 (± 0.17)	0.35 (± 0.02)	1.88 (± 0.20)	sm (m)	2.11 (± 0.11)	0.33 (± 0.03)	2.08 (± 0.25)	sm	2.22 (± 0.27)
	0.41 (± 0.07)	1.48 (± 0.44)	m (sm)	2.21 (± 0.11)	0.36 (± 0.02)	1.82 (± 0.12)	sm (m)	2.12 (± 0.07)	0.33 (± 0.04)	2.07 (± 0.42)	sm (m)	2.19 (± 0.25)
14	0.37 (± 0.04)	1.74 (± 0.26)	sm (m)	2.08 (± 0.24)	0.40 (± 0.05)	1.51 (± 0.34)	m (sm)	2.11 (± 0.09)	0.40 (± 0.03)	1.52 (± 0.16)	m	1.97 (± 0.09)
	0.38 (± 0.05)	1.64 (± 0.28)	m (sm)	2.01 (± 0.24)	0.40 (± 0.06)	1.54 (± 0.37)	m (sm)	2.10 (± 0.09)	0.42 (± 0.02)	1.41 (± 0.13)	m	1.98 (± 0.12)
15	0.44 (± 0.04)	1.29 (± 0.20)	m	1.96 (± 0.11)	0.39 (± 0.05)	1.58 (± 0.32)	m (sm)	1.79 (± 0.25)	0.46 (± 0.02)	1.15 (± 0.07)	m	1.73 (± 0.27)
	0.41 (± 0.04)	1.43 (± 0.20)	m	1.84 (± 0.30)	0.40 (± 0.06)	1.52 (± 0.37)	m (sm)	1.78 (± 0.22)	0.46 (± 0.01)	1.19 (± 0.07)	m	1.70 (± 0.22)
16	0.42 (± 0.04)	1.38 (± 0.20)	m	1.79 (± 0.31)	0.44 (± 0.02)	1.30 (± 0.12)	m	1.59 (± 0.14)	0.49 (± 0.03)	1.06 (± 0.14)	m	1.62 (± 0.22)
					0.42 (± 0.02)	1.17 (± 0.09)	m	1.49 (± 0.17)	0.48 (± 0.00)	1.08 (± 0.02)	m	1.60 (± 0.19)
17									0.47 (± 0.01)	1.11 (± 0.05)	m	1.56 (± 0.20)

Table S2 Summary of the available data per specimen.

	St #	specimen	sex	modal chromosome number	number of supernumerary chromosomes	Assigned karyomorph	Heterchromatin distribution
TUNU I	889	JM14	F	30	2	C	11p
	889	JM15	M	29	1	B	10q
	889	JM16	F	30	2	C	1q 10p
	889	JM17	M	30	2	C	1p1q
	889	JM18	F	30	2	C	1q 10q
	889	JM19	M	30	2	C	1q 10q 11p
	889	JM25	F	28	0	A	11p
	889	JM27	juv	30	2	C	1q 10q
	889	JM28	M	28	0	A	1q 10p
	889	JM30	M	30	2	C	10p
	892	JM20	M	31	3	D	1q 10q 11p
	892	JM21	M	33	5	F	10q
	892	JM23	F	30	2	C	1q
TUNU II	644	JM 91	F	30	2	C	11p
	644	JM 92	M	30	2	C	1q 10q 11p
	644	JM 94	F	31	3	D	1q 10q 11p
	644	JM 95	M	33	5	F	1q
	644	JM 96	F	33	5	F	1p 1q 11p
	644	JM 97	M	31	3	D	1q 11p
	644	JM 101	M	32	4	E	10p
	644	JM 103	M	31	3	D	11p
	664	JM 105	M	31	3	D	10q 11p
	644	JM 106	F	32	4	E	11p
TUNU IV	8	JM 428	F	31	3	D	1q 11p
	13	JM 456	juv F	31	3	D	1q 11p
TUNU V	ST 001	JM506	F	30	2	C	1p 1q 11p
	ST 006	JM 555	M	31	3	D	1q
	ST 006	JM 556	F	29	1	B	11p
	ST 006	JM 557	M	31	3	D	1q 10p 11p
	ST 006	JM 559	F	31	3	D	1p 1q 11p
	ST 006	JM 560	F	28	0	A	1q
	ST 009	JM 570	F	30	2	C	11p
	ST 009	JM 571	M	31	3	D	11p
